# The Significance of Demographic Variables on Psychosocial Health from the Early Stage and Nine Months after the COVID-19 Pandemic Outbreak. A Cross-National Study

**DOI:** 10.3390/ijerph18084345

**Published:** 2021-04-20

**Authors:** Amy K. Østertun Geirdal, Daicia Price, Mariyana Schoultz, Hilde Thygesen, Mary Ruffolo, Janni Leung, Tore Bonsaksen

**Affiliations:** 1 Department of Social Work, Faculty of Social Sciences, Oslo Metropolitan University, 0167 Oslo, Norway; 2 School of Social Work, University of Michigan, Ann Arbor, MI 48109, USA; daiciars@umich.edu (D.P.); mruffolo@umich.edu (M.R.); 3 Department of Health and Life Sciences, Northumbria University, Newcastle upon Tyne NE1 8ST, UK; Mariyana.schoultz@northumbria.ac.uk; 4 Department of Occupational Therapy, Prosthetics and Orthotics, Faculty of Health Science, Oslo Metropolitan University, 0167 Oslo, Norway; hilde.thygesen@oslomet.no; 5 Faculty of Health Studies, VID Specialized University, 4306 Sandnes, Norway; tore.bonsaksen@inn.no; 6 Faculty of Health and Behavioural Science, University of Queensland, Brisbane, QLD 4072, Australia; j.leung1@uq.edu.au; 7 Department of Health and Nursing Science, Faculty of Social and Health Sciences, Inland Norway University of Applied Sciences, 2418 Elverum, Norway

**Keywords:** COVID-19, coronavirus, psychosocial health, mental health, quality of life, well-being, loneliness, cross-national study

## Abstract

This cross-national study explored stability and change in mental health, quality of life, well-being and loneliness during the early stage and nine months after the implementation of COVID-19 pandemic social distancing measures and periodic lockdowns as adjusted by demographic variables. In the USA, the UK, Australia and Norway, 7284 individuals responded to the invitation to take part in two cross-sectional web-based surveys (April and November 2020), including questions about sociodemographic variables and psychosocial outcomes. Independent *t*-tests and generalized linear models (GLM) and estimated marginal means were used to analyze differences between subgroups and countries, multiple linear regression analyses were conducted on the psychosocial outcome measures by demographic variables and time in each country and mean responses presented by time after adjusting for all demographic variables in the model. Age, gender, civil status, education, employment, place of work and living area were all significant factors for psychosocial health across the countries. Differences in mental health, quality of life, well-being and loneliness were found between the countries in both April and November 2020, while time did not contribute to reducing the toll in any of the four countries over the nine-month period.

## 1. Introduction

During the COVID-19 pandemic, the main policy for public behavior has been periodic lockdowns and social distancing [1] including recommendations of an appropriate distance between individuals outside one’s own household. To prevent viral spread, people were asked to stay at home as much as possible and only do “necessary” travels. Businesses defined as non-essential, for example, gyms and furniture stores were temporarily shut down at different points throughout the pandemic. Schools and universities have been either closed for in-person classroom learning or moved to distance learning, remote teaching and online instruction formats [2]. Many people have transitioned to working remotely [3], and those working at their workplaces, e.g., in healthcare, have had to take great precautions and follow strict infection control guidelines [4]. 

A review article based on 19 cross-sectional studies that focused on the association between the COVID-19 pandemic and mental health revealed that being female was associated with a higher degree of mental distress than among men [5] and that being older was associated with experiences of better mental health. The finding is in line with earlier studies, e.g., Carstensen et al. [6] and Neubauer et al. [7] showed that older people in general report fewer negative emotions than younger people, and stressful events are perceived as less unpleasant. According to Lõwe et al. [8], older people tend to score lower on depression and anxiety measures. 

According to Xiong et al. [5], the COVID-19 pandemic represents a threat to mental health worldwide, independent of a country’s status as low-, middle-, or high-income. This is in line with a previous study [9] that found that in a number of western countries, COVID-19 bears a significant impact on psychosocial health including a higher level of emotional distress, poorer quality of life and well-being and increased feeling of loneliness. Other studies, as summarized in a review study [10], measured well-being and psychological distress in the general public and confirmed worse psychological well-being and higher scores of psychological distress including increased levels of depression and anxiety and high levels of post-traumatic stress disorder (PTSD) [10,11].

In the initial phase after the COVID-19 outbreak, Wang et al. [12] performed a longitudinal study looking at the impact of COVID-19 on mental health in the general population (January/February (T1) and February/March (T2) 2020) and found stable levels of psychological distress between T1 and T2. British longitudinal studies based on the same population before and after the outbreak of COVID-19 concluded that prolonged deterioration in mental health occurred after the COVID-19 outbreak [13,14]. 

However, most studies with a focus on mental health, quality of life and well-being due to the COVID-19 outbreak are cross-sectional studies that do not address how time may impact psychosocial health. Based on this and the lack of knowledge related to the importance of time on psychosocial health, we performed two consecutive cross-sectional studies that allowed for a comparison between timepoints using appropriate statistical tools.

### Study Aim

The aim of this study was to explore stability and change in mental health, quality of life, well-being and loneliness from the early stage and nine months after the COVID-19 pandemic outbreak as adjusted by demographic variables in two cross-national samples.

## 2. Material and Methods

### 2.1. Design and Procedures

The study used a cross-sectional survey design; the survey was distributed through social media in each of the involved countries in April (T1) and November (T2) 2020. The same survey was used at both timepoints with minor adjustments for the second administration of the survey [3,9]. The landing sites for the survey were established at the researchers’ universities: OsloMet—Oslo Metropolitan University, Norway; University of Michigan, USA; Northumbria University, UK; and the University of Queensland, Australia. The initiator of the project was A.K.Ø.G. from OsloMet. Due to ethical considerations and permissions in each of the countries, each country had its own project lead. The survey was simultaneously codeveloped by the researchers in two languages: Norwegian and English. Language and cultural differences were considered during the survey development process, meaning that the countries’ phrasing of each item conveyed the same meaning when considering culturally embedded meanings of words and phrases.

### 2.2. Inclusion and Exclusion

To be included in the study, participants had to be 18 years or older, understand Norwegian or English, live in Norway, the USA, the UK or Australia and be able to access the electronic survey. There were no exclusion criteria. Altogether, 7284 individuals participated by responding to the online form. 

### 2.3. Measures

#### 2.3.1. Sociodemographic Characteristics and Time of Survey

Sociodemographic variables included country of residence (NO, USA, UK, AUS); gender (male (1), female (2)); age (18–24 years (1), 25–34 years (2), 35–44 years (3), 45–54 years (4), 55–64 years (5), 65–74 years (6), over 75 years (7)); highest completed level of education (Bachelor’s degree or below (1), Master’s/Doctoral degree (2)); civil status (living with a spouse or partner, no (0), yes (1); having children, yes (1), no (0)); employment (full-time (1) or part-time employment (2)), place of employment/workplace (healthcare (1), trade industry (2), construction industry (3), education (4), public safety (5), other (6)) and living area (rural or farming area (1), town/suburb (2), city (3)) (Table 1).

#### 2.3.2. Mental Health, Quality of Life, Well-Being and Loneliness

General Health Questionnaire 12 (GHQ-12) is widely used as a self-report measure of mental health [15,16,17]. A large number of studies in the general adult, clinical, work and student populations have provided support for its validity across samples and contexts [16,18,19,20,21,22,23,24] and translated from English to several other languages, including Norwegian [25,26]. Six items of the GHQ-12 are phrased positively (e.g., ‘able to enjoy day-to-day activities’) and six—negatively (e.g., ‘felt constantly under strain’). The person indicates the degree to which the item content was experienced during the two preceding weeks using four response categories (‘less than usual’ (0), ‘as usual’ (1), ‘more than usual’ (2) or ‘much more than usual’ (3)); score range, 0–36. Positively formulated items are recoded prior to analysis. Higher scores indicate poorer mental health (more psychological distress). Case-level scores (the person indicating ‘more than usual’ or ‘much more than usual’ on at least four of the 12 items) indicate a level of emotional distress where treatment may be needed [27]. Cronbach’s alpha ranged between 0.88–0.92 in the current samples across the countries, and the Pearson correlation coefficient between the questions and the total value showed significance <0.05 indicating the questionnaire’s validity in the total sample and across the four national samples. 

Cantril’s self-anchoring ladder (CL) is a self-administered overall quality of life (QoL) questionnaire with one question, ‘How is your life?’, asking the person to rate his or her present experience of life on a scale anchored by their own identified values [28] and used when comparing satisfaction with life between groups and populations [29,30,31,32,33]. The response alternatives are between 0 and 10 with 0 = worst possible QoL and 10 = best possible QoL [28]. The cut-off score was chosen at 6 and above for good overall quality of life [28]. The CL has been reported to have good validity and stability and reasonable reliability [34,35,36,37]. 

Psychosocial well-being (PSW) assesses an individual’s psychological experience of well-being and consists of ten items. The measure includes five positive and five negative statements with item scores ranging between 1 (best well-being) and 5 (worst well-being) [38]. Cronbach’s alpha was excellent, ranging from 0.89–0.90 across the country samples. Validity tests in the current questionnaire indicated good validity and it is good evidence to propose a one-factor solution to the PSW scale as used in this study [9] and consistent with previous validation studies [38].

The Loneliness Scale [39] consists of six statements, all of which are rated from 0 (‘totally disagree’) to 4 (‘totally agree’). It measures two different aspects of loneliness, ‘emotional loneliness’ and ‘social loneliness’. Previous factor analyses found the six statements to load on two different factors, and therefore they should be treated as constituting two different scales reflecting the two different aspects of loneliness [39,40]. For both scales, the score range is 0–12 with higher scores indicating more loneliness. Using a one-factor solution to measure overall loneliness may also be appropriate (score range, 0–24), depending on the level of conceptual nuance required. In this study, only overall loneliness was included in the analysis. Higher sum scores indicated higher overall loneliness. Cronbach’s alpha was good, 0.80 for overall loneliness. 

### 2.4. Statistical Analysis

Mental health, overall quality of life, psychosocial well-being and overall loneliness were calculated for each included demographic subgroup (gender, age, civil status, having children, education level, employment, workplace and place of living) within each country at each timepoint. 

Depending on the number of group categories, group differences were examined using the one-way analysis of variance (ANOVA) and the independent *t*-test. The Pearson correlation coefficient *r* was used to assess the strength between categorical data. 

In order to examine whether changes in mental health, overall quality of life, psychosocial well-being and loneliness were different between the four countries, the time × country variable was computed in SPSS and entered as a predictor (Step 2) in the regression analysis. The sociodemographic variables gender, age, civil status, having children, education and employment were included in Step 1. In cases of significant interaction effects (time × country), the subsequent regression analyses were performed separately for each country.

Generalized linear models (GLM) were used to conduct multiple linear regression analyses on psychosocial measures (GHQ, CL, PSW, Loneliness) by demographic variables and time (November vs. April) in each country. Estimated marginal means were used to present mean responses by time after adjusting for all demographic variables in the model. Unstandardized beta (B) and standard error (ES) were reported for measures of the effect of associations between the independent and dependent variables. Statistical significance was set at <0.05. Missing values were handled by case-wise deletion.

### 2.5. Ethics

The data collected in this study were anonymous. The researchers adhered to all relevant regulations in their respective countries concerning ethics and data protection. The study was approved by OsloMet (20/03676) and the regional committees for medical and health research ethics (REK; ref. No. 132066) in Norway, reviewed by the University of Michigan’s Institutional Review Board for Health Sciences and Behavioral Sciences (IRB HSBS) and designated as exempt (HUM00180296) in the USA, by Northumbria University Health Research Ethics Committee (HSR1920-080) in the UK, as well as in Australia (HSR1920-080 2020000956). 

## 3. Results 

### 3.1. Participants

The total sample from Survey 1 (T1) was 3810 [3,9], including 771 (20%) indididuals from Norway, 1393 (37%) from the USA, 1373 (36%) from the UK and 210 (7%) from Australia [3,9].

The participants included in Survey 2 (T2) were 3474 individuals from Norway (*n* = 547, 16%), the USA (*n* = 2130, 61%), the UK (*n* = 640, 18%) and Australia (*n* = 157, 4%); altogether, 7284 individuals from both surveys. Overall, there were more women than men in the total sample (77% versus 20%, respectively) and the individuals who responded were spread across age groups. Sixty percent lived with a spouse or partner, 60% had children and 64% had a Bachelor’s degree or lower levels of education. Sixty-eight percent had either a full-time or a part-time job, and of these individuals, 18% worked in healthcare, 4%—in trade industry, 14%—in education and 7%—in other critical functions in the society (public safety, police, fire services, etc.); 24% reported other areas of work. Eleven percent lived in rural areas, 51%—in a town or a suburb, while 38% lived in a city.

### 3.2. Mental Health, Overall Quality of Life, Well-Being and Loneliness at T1 and T2 Combined

Table 2 displays the levels of psychosocial health according to subgroups. Women reported a significantly higher level of emotional distress (*p* < 0.001) and poorer well-being (*p* < 0.001) than men. The lower the age of the individual, the higher the level of emotional distress (*p* < 0.001), poorer quality of life (*p* < 0.001) and well-being (*p* < 0.001) and higher the feeling of loneliness (*p* < 0.001). Participants living together with a spouse or partner reported less emotional distress (*p* < 0.001), better quality of life (*p* < 0.001) and well-being (*p* < 0.001) and less loneliness (*p* < 0.001) than their counterparts. The same overall pattern was found among those who had children (*p* < 0.001 for all outcomes, respectively), had a Master’s degree or higher education (*p* < 0.01 for CL, *p* < 0.001 for GHQ, PSW and Loneliness, respectively) and were in full-time employment (*p* < 0.001 for GHQ and PSW, *p* < 0.01 for Loneliness). People working in trade industry exhibited the highest level of emotional distress (*p* < 0.001) and felt most lonely (*p* < 0.01), while those living in rural or farming areas reported less emotional distress (*p* < 0.001), better quality of life (*p* < 0.001) and a higher level of well-being (*p* < 0.001) than those living in towns/suburbs or cities.

### 3.3. Mental Health, Overall Quality of Life, Well-Being and Loneliness in and between Countries and Time

The levels of emotional distress when measured as mean and standard deviation were higher in the Norwegian sample (*p* < 0.001) and at the same level in the three other countries in November 2020 compared to April 2020 (Table 3). However, fewer individuals had scores equal to the case level in November compared to April in all the four countries (*p* < 0.001). Alternatively, a higher number of individuals reported poorer quality of life on the case level for quality of life in November compared to April (*p* < 0.001, all countries), and in line with this, the mean scores of quality of life (CL) were significantly reduced in all countries (*p* < 0.001) except the UK. Psychosocial well-being was reported significantly poorer in the UK and Norway in November compared to April (*p* < 0.001), while the Australian and USA samples reported significantly better well-being (*p* < 0.05 and *p* < 0.001, respectively) in the same timeframe (Table 3). All the countries’ samples except the Australian one reported a significantly higher level of loneliness between the timepoints. Significant differences between the countries were found for all outcomes in both April and November.

### 3.4. Regression Analysis

When assessing whether changes in the outcomes across the two timepoints differed between the countries, regression analysis showed that the time by country interaction effects were significant for all outcomes (GHQ *p* < 0.001, CL *p* < 0.001, PSW *p* < 0.001, Loneliness *p* < 0.001) when controlled for the included demographic variables as described above (data not shown).

### 3.5. Multiple Linear Regression

When the generalized linear models (GLM) were used to conduct multiple linear regression analyses on the psychosocial measures (GHQ, CL, PSW, Loneliness) by demographic variables and time (November vs. April) in each country, both demographic variables and time of survey explained a significant effect of the values of GHQ, CL, PSW and Loneliness (Table 4). 

The analysis showed that age and civil status were the independent factors that showed the strongest unique effects across all the countries. 

When controlling for all demographic variables, time had a significant contribution on the higher level of emotional distress and poorer well-being in the Norwegian sample (*p* < 0.01 and *p* < 0.01, respectively) and of poorer well-being in the UK samples (*p* < 0.01) (Table 4). 

**Figure 1:**
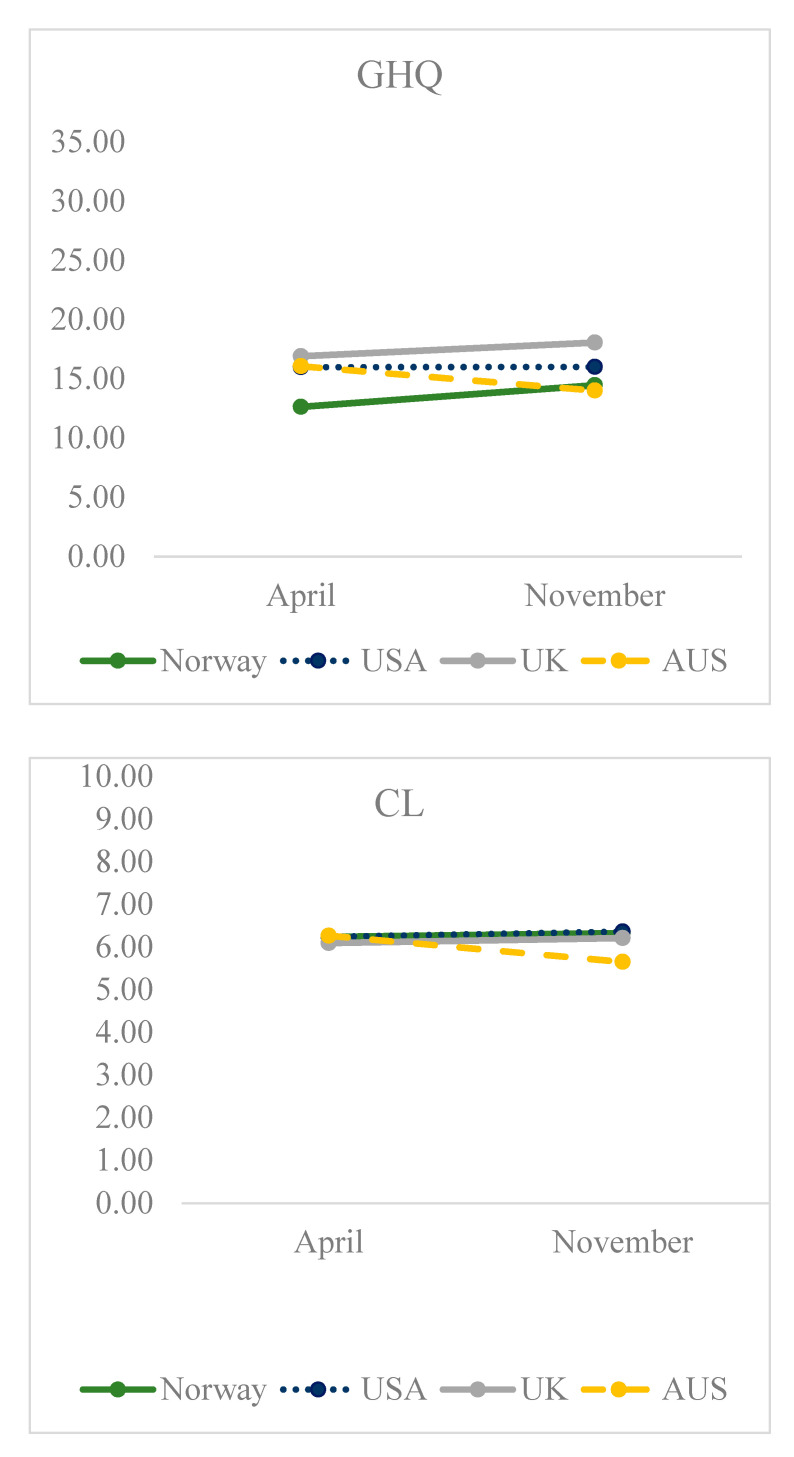

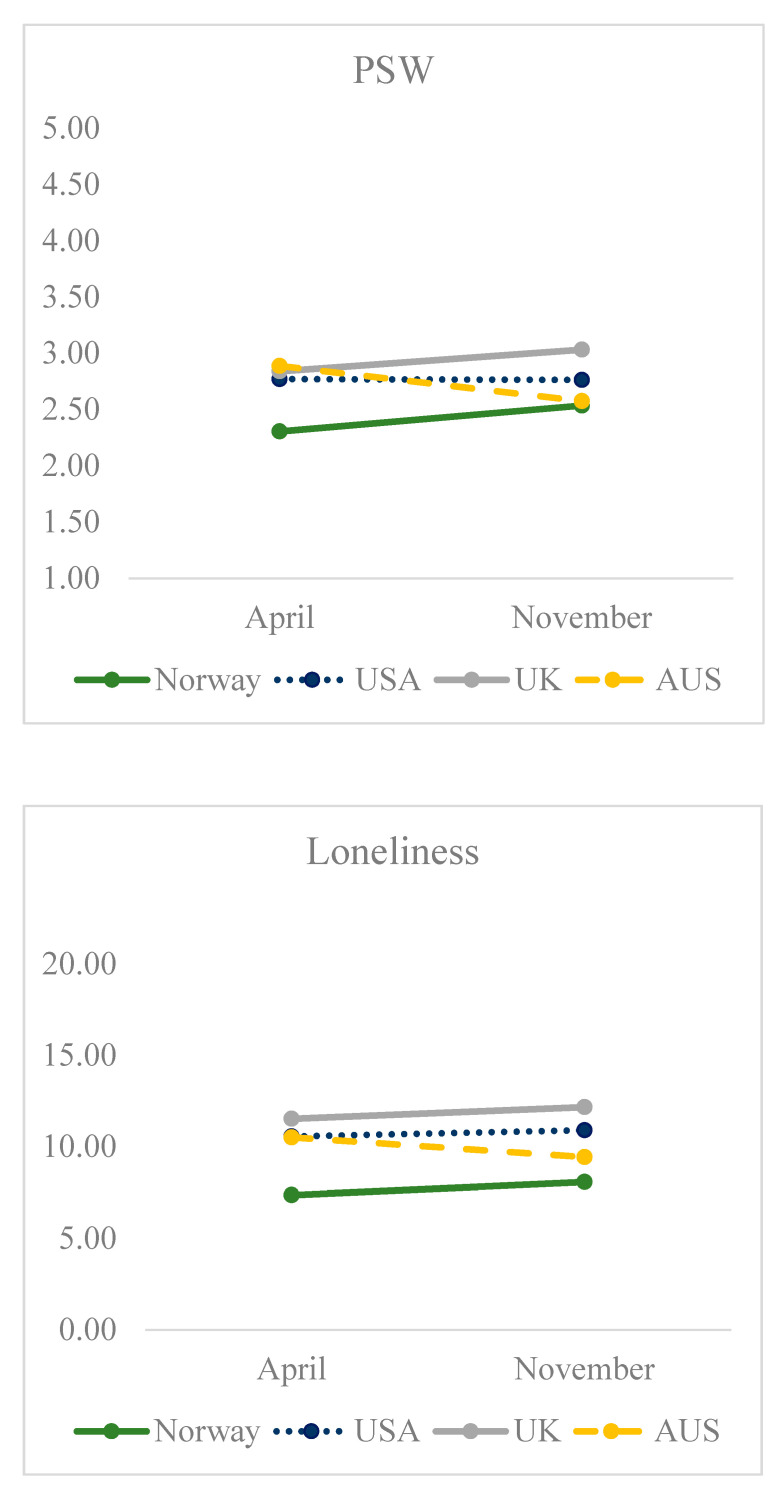
Adjusted means. See Section 2.3.2. Mental health, quality of life, well-being and loneliness in the measurement units used.

The estimated marginal mean responses by time after adjusting for all demographic variables in the model are shown in Figure 1.

## 4. Discussion

The most important findings in this paper were that the levels of emotional distress, quality of life, well-being and loneliness either got poorer or were stable but at a poor level over time. The Norwegian sample reported a higher level of emotional distress, decreased quality of life, poorer well-being and higher level of loneliness over the time on the group level. All outcome variables were mainly stable in the three other countries in the same time period. Nonetheless, the Norwegian sample had significantly better scores in all outcome measures than the comparing countries. 

In ordinary times, a high level of emotional distress (GHQ) may indicate reduced functional impairment equal to a mental disorder. However, in this particular study, it is clear that these findings are associated with the ongoing COVID-19 pandemic. Even if the level of caseness is reduced compared to the reports in April 2020, it is still much higher than the 20% within a year in all countries due to mental distress before the COVID-19 outbreak [41,42,43,44,45]. It is of interest though that the caseness is reduced throughout the samples. Even if it is to be assumed that social distancing still has an important impact on mental health, it might be that people have become more used to the situation and are adapting to using different coping strategies. 

According to the OECD [46], the average level of quality of life for western countries, including those in this study, was 7.4 in 2018 as measured with CL. However, life is dynamic and changing, and it seems that the situation has gone from better to worse not only when compared to the reported levels in general populations in 2018, but also between the results in April and November 2020 in our study. While 27% (Norway), 30% (USA), 36% (UK) and 24% (Australia) reported caseness for poorer quality of life in April, the findings for the same countries were 38%, 56%, 51% and 59%, respectively, in November. A cut-off score ≤5 is defined as suffering due to the experienced overall quality of life [28], meaning the experienced overall quality of life is moderate or inconsistent. This means that people are struggling in their present situation and may have negative thoughts and views about the future. The pandemic’s social distancing protocols had been in place for several months by the Survey 2 measurement, and the reduced quality of life reported reflects individuals struggling with uncertainty about the future. The reduced quality of life may be associated with the social distancing protocols that reduced social relationships and meetings with colleagues and friends outside one’s household, promoted engagement in remote work and implemented limitations on travelling. On the other hand, participants might have gotten accustomed to the changed situation that in some ways may have reduced the initial heavy burden of mental distress experienced early in the pandemic. In line with this, the difference between the countries when it comes to reported well-being might be understood as an adaptation to the ongoing pandemic. At the same time, it is to be expected that most people will over time return to what was known as a normal level of both mental health and experienced quality of life. 

The fact that the Norwegian sample reported a higher level of emotional distress in November than in April does not remove the significant differences between the countries in November. While the Norwegian sample still reported significantly less emotional distress than the other countries, it might have been attributed to cultural differences and the level of supports provided in the welfare systems. Differences in perceived trust in how political systems, government and health authorities handle the pandemic and meet challenges due to the COVID-19 pandemic may also contribute to these results similar to what is described in previous research [47].

Prior research, e.g., by Rosenfiled et al. [48], underscored that the gender status does not necessarily have a significant impact on overall mental health, but that it may be related to different kinds of mental health issues. Women, for example, report more internalizing diseases like depression and anxiety, while men are more likely to report more externalizing disorders like antisocial behavior and drug abuse [48,49]. These prior findings are consistent with the current study since the GHQ measures of psychological distress include anxiety and depression. In addition, our findings are in line with the review study based on 19 cross-sectional studies measuring mental health related to gender in the time of COVID-19 [5]. The review study underscores that identifying as female is associated with more vulnerability for mental distress than identifying as male. 

Age and civil status in particular were variables associated with significant contribution to emotional distress, quality of life, well-being and loneliness across the countries. Higher age seems to be a buffer against higher emotional distress, poorer quality of life, well-being and loneliness. The impact on mental health seems to take a higher toll on younger people and this finding is in line with research that points out that younger people have more negative emotions and experience stressful events as more unpleasant than older people [6,7], and older people in general score lower on depression and anxiety [8]. An explanation for the results in the current study might be that young people normally look forward, and in the present situation they may feel their daily lives and the future are set on hold or crumbling away due to loss of social interactions. According to Mykle-stad et al. [50], support from friends is generally associated with lower levels of distress among adolescents. Older people, on the other hand, need, perhaps, to achieve less, they can look back to good memories and joy and know through life experience that most stressful times will end, even if it takes time. Older people have a variety of tools in the toolbox to manage a particular situation of stress when compared to the younger population. Another possible explanation might be that in the countries studied, older people are the first in line for vaccination and on a faster track to resuming a normal life. However, pre-pandemic studies suggested that social isolation and older age were associated with loneliness [51], which is seen as a key public health issue [52,53]. On the other hand, previous research also showed that the number of contacts is not important for psychological distress among elderly people, but social support is associated with decreased emotional distress [54]. 

Living with a spouse or a partner was associated with less emotional distress, better quality of life and well-being and less loneliness and could simply be explained by the support of having a close person to confide in, and that living with a spouse or a partner is a buffer against psychosocial stress. This study did not examine mental health with regard to living with other people or a pet. It is to be assumed that for some of those not living with a spouse or a partner living with someone or having a pet would be of big importance. 

Respondents with a Bachelor’s degree or below exhibited higher levels of emotional distress, poorer quality of life and well-being and more loneliness than those with a Master’s degree or higher education. However, when controlled for other demographic variables, education had a significant contribution to mental health, well-being and loneliness in the Australian sample, and to well-being and loneliness in the USA sample. While education did not contribute to any significant outcomes in the Norwegian sample, it could be explained by an overall higher educational level in the Norwegian population than, for example, in the sample from the USA. An alternative speculation may be that health inequalities by education level are less pronounced in Norway. On the general level, education is assumed to buffer the effect of a disability, in this case associated with a global situation. A reason for this might be the more educated are more equipped, including cognitive skills, to cope with the consequences of a particular cause of stress [55].

The individuals who were employed full-time exhibited less emotional distress, better well-being and less loneliness than those who were employed part-time. In the multiple hierarchical regression, employment had a significant unique contribution to reduced emotional distress in all the countries over time, which is in line with former cross-sectional studies [3] and former studies underscoring that employment, in general, is associated with improved mental health and quality of life [56]. Having a job often means that people have a social network and experience social inclusion through their work. In line with the findings, it might also be understood that higher levels of employment may result in better psychosocial health over time.

Healthcare workers exhibited a high level of emotional distress, reduced well-being and loneliness, which is consistent with former studies [4], but in this study, even higher scores on these measures were reported by those employed in the trade industry. While healthcare workers are constantly exposed to the increased risk of physical contact with infections and have had to take greater precautions and follow strict infection control protocols, trade workers may not have had adequate infection control equipment and supports apart from hand washing, antibacterial cleaning practices in the work setting, masks and keeping distance. Another reason for the higher scores in the trade industry may be associated with the uncertainty about the potential loss of jobs due to closedowns. Teachers, which is another group with high scores, have very limited possibilities for keeping distance in the workplace and may have increased concerns of infection, sickness and quarantine.

Interestingly, living in a rural or farming area had a positive impact on emotional distress, quality of life and well-being. This does not necessarily mean that there were no infections in these areas, but it may suggest that the toll of the pandemic was smaller in rural areas than in suburbs or cities 

Time contributed significantly to emotional distress (GHQ). This may be understood as the Norwegian sample was the only country with significantly different scores between April and November 2020. In the countries with higher but stable scores, time had no significant impact. On the other hand, it is to be concluded that, overall, time did not contribute to lowered levels and reduce the toll on mental health, quality of life, well-being or loneliness in any of the four countries.

The pandemic is still causing social distance, quarantine, and isolation protocols in many places around the world. More research is needed on how COVID-19-related risk variables and concerns are associated with time so that appropriate support systems could be put in place to improve public psychosocial health. In light of this, health and social workers should be aware of the importance of using professional skills to promote better psychosocial health in meeting individuals with needs, such as young people and those living alone. For example, and as underscored by Dorado et al. [57], social work interventions are thought to have a key role when it comes to improving psychosocial factors such as well-being among individuals suffering from COVID-19-produced consequences. Further research could have a more specific focus on how health workers and social workers engage people in such a need. Understanding how risk factors present during the COVID-19 pandemic are associated with an individual’s mental health, quality of life, well-being and loneliness warrants future research as well. 

### Study Strength and Limitations

The strength of this study was the cross-national comparison and the relatively high number of respondents. However, some limitations should be taken into consideration. First, the data were collected using cross-sectional online surveys, which means assumptions about causations cannot be made, only associations. Secondly, the respondents received invitations to participate through social media, and the responses thereby do not include individuals that do not use social media. This may limit the ability to generalize the results to the general population in the four countries included in the study. The sample included respondents from all age groups, but fewer from the oldest age group, as well as more women than men. This may represent a skewed population. To avoid this, in future studies, more sophisticated sampling methods could be used. Third, our findings imply that social distancing policies are significant for psychological distress, quality of life, well-being and loneliness. The degree of disease outbreak and social distancing policies differed between the countries and the states in, for example, the USA and may have included additional factors on individual levels, as well as on the regional and country level. For example, the restriction levels in the four countries at T1 and T2 varied between moderate and high levels, including closures of schools and universities, variations of “stay at home” orders and closedowns, restrictions regarding gatherings of people from different households, closed borders, directions to stay at home other than for essential reasons and no unnecessary travel. Both in April and November, the number of infected people and deaths varied between the countries with the USA and the UK having higher rates than Norway and Australia, and both surveys, T1 and T2, took place before the vaccination rollouts. These issues could have influenced the respondents’ answers. 

## 5. Conclusions

Overall, significant differences in psychosocial health across Norway, the USA, the UK and Australia emerged in this study. Individuals in the younger age groups struggled more than the older groups with mental health, quality of life, well-being and loneliness. Being female, not married or cohabiting, lower level of education, unemployed, working in healthcare or the trade industry and living in urban areas are all factors significantly associated with poorer psychosocial health across the countries. However, when adjusting for months into the pandemic (time) and other demographic variables, age and living together with a spouse or a partner contributed most to the psychosocial outcomes in all the countries. Differences in mental health, quality of life, well-being and loneliness were found between the countries in both April and November 2020. The toll of the COVID-19 pandemic on mental health did not reduce in any of the four countries between April and November 2020.

## Figures and Tables

**Table 1:** Overview of the sociodemographic characteristics.

Sociodemographic Characteristics	Answering Options
Country of residence	NO USA UK AUS
Gender	Women Men Other
Age	<24 years 25–34 years 35–44 years 45–54 years 55–64 years 65–74 years >75 years
Civil status	Married/cohabiting Not married/cohabiting
Having children	Yes No
Education	≤Bachelor ≥Master
Employment	Full-time Part time
Place of work	Healthcare Trade industry Education Public safety, police, fire services, etc. Other
Living area	Rural or farming area Town/suburb City

Time of survey included April 2020 (1) and November 2020 (2).

**Table 2:** Mental health, overall well-being, quality of life and loneliness in sample subgroups nine months after the COVID-19 outbreak in the total sample from April and November 2020.

			GHQ		CL		PSW		Loneliness	
		*N* = 7284	Mean (SD)	*p*	Mean (SD)	*p*	Mean (SD)		Mean (SD)	
Demographic variables	Gender			<0.001		0.5		<0.001		<0.001
	Women	5580	16.8 (6.6)		6.2 (2.2)		2.8 (0.8)		10.5 (4.9)	
	Men	1489	14.8 (7.1)		6.3 (2.5)		2.6 (0.9)		10.2 (5.3)	
	Other	215	18.45 (8.2)		6.1 (2.2)		3.1 (0.9)		12.2 (5.3)	
	Age			<0.001		<0.001		<0.001		<0.001
	<24 years	655	19.3 (7)		5.8 (2.4)		3.1 (0.8)		11.8 (4.7)	
	25–34 years	1478	18.1 (6.5)		5.9 (2.2)		2.9 (0.8)		10.7 (4.7)	
	35–44 years	1327	16.9 (6.8)		6.1 (2.2)		2.8 (0.8)		10.3 (5.)	
	45–54 years	1299	15.9 (6.6)		6.3 (2.3)		2.7 (0.8)		9.9 (5)	
	55–64 years	1112	15.1 (6.6)		6.5 (2.3)		2.6 (0.8)		9.6 (4.8)	
	65–74 years	830	13.4 (6.1)		6.8 (2.4)		2.4 (0.8)		9.1 (4.6)	
	>75 years	182	12.8 (5.2)		6.6 (2.4)		2.3 (0.8)		8.6 (4.5)	
	Civil status			<0.001		<0.001		<0.001		<0.001
	Married/cohabiting	4379	15.8 (6.5)		6.4 (2.2)		2.6 (0.8)		9.7 (4.8)	
	Not married/cohabiting	2638	17.3 (7.2)		5.9 (2.4)		3 (0.9)		11.6 (5.1)	
	Having children			<0.001		<0.001		<0.001		<0.001
	Yes	4344	15.4 (6.5)		6.5 (2.8)		2.6 (0.8)		9.8 (4.9)	
	No	2940	17.6 (6.9)		5.9 (2.3)		3 (0.8)		10.7 (4.8)	
	Education			<0.001		<0.01		<0.001		<0.001
	≤Bachelor	4644	16.8 (7.1)		6.1 (2.4)		2.8 (0.9)		10.7 (4.9)	
	≥Master	2570	15.7 (6.2)		6.4 (2.2)		2.6 (0.8)		9.3 (4.7)	
	Employment			0.001		0.9		<0.001		<0.01
	Full-time	3580	16.2 (6.5)		6.2 (2.2)		2.7 (0.8)		10.3 (4.9)	
	Part time	1411	17.3 (6.7)		6.1 (2.3)		2.9 (0.8)		10.8 (5.1)	
	Place of work			<0.001		0.4		<0.05		<0.01
	Healthcare	882	16 (6)		6.3 (2.3)		2.7 (0.8)		9.5 (4.6)	
	Trade industry	176	17.2 (7.4)		5.8 (2.6)		2.9 (0.9)		10.7 (5.2)	
	Education	697	16.9 (6.2)		6.3 (2.1)		2.8 (0.7)		10.2 (4.9)	
	Public safety, police, fire services, etc.	370	14.7 (6.2)		6.3 (2.2)		2.7 (0.8)		10.5 (5.2)	
	Other	1379	16.7 (6.9)		6.2 (2.2)		2.8 (0.8)		10.4 (4.9)	
	Living area			<0.001		<0.001		<0.001		0.9
	Rural or farming area	794	15.4 (6.7)		6.4 (2.3)		2.6 (0.9)		10 (5.2)	
	Town/suburb	3672	16.4 (6.8)		6.3 (2.3)		2.7 (0.8)		10.3 (4.8)	
	City	2744	16.9 (6.9)		6.0 (2.3)		2.8 (0.8)		10 (4.9)	

**Table 3:** Comparison of mental health, overall quality of life, psychosocial well-being and loneliness between the national samples and between the countries in April and November 2020.

		Norway			USA			UK			Australia			Total	
		April N = 771	November *N* = 547	*p*	April *N* = 1393	November *N* = 2130	*p*	April *N* = 1373	November *N* = 640	*p*	April *N* = 273	November *N* = 157	*p*	*N* = 3810 Differences between countries April 2020	*N* = 3474 Differences between countries November 2020
GHQ	Mean (SD)	13.7 (6.5)	14.9 (7)	<0.001	16.3 (6)	16.4 (6.6)	0.10	18.5 (7.6)	18.6 (7)	0.5	16.1 (6.8)	15.2 (6.9)	0.16	<0.001	<0.001
	Case (*N*/%)	375/49	242/44	0.06	950/70	1033/56	<0.001	1015/74	373/68	<0.01	172/63	55/42	<0.001	<0.001	<0.001
CL	Mean (SD)	6.7 (1.9)	6.1 (2.4)	<0.001	6.6 (2.1)	6.3 (2.8)	<0.05	6.1 (2.3)	6 (2.2)	0.12	6.8 (2.2)	5.8 (2.4)	<0.001	<0.05	<0.05
	Case (*N*/%)	204/27	210/38	<0.001	414/30	1186/56	<0.001	498/36	324/51	<0.001	64/24	92/59	<0.001	<0.001	<0.001
PSW	Mean (SD)	2.2 (0.7)	2.6 (0.9)	<0.001	2.9 (0.4)	2.7 (0.9)	<0.001	2.8 (0.3)	3 (0.8)	<0.001	2.9 (0.3)	2.7 (0.9)	0.05	<0.001	<0.001
Loneli-ness	Overall loneliness (Mean/SD)	7.8 (4.6)	8.4 (5)	<0.01	10.2 (4.5)	10.8 (4.9)	<0.001	11.0 (4.8)	11.5 (4.5)	<0.05	9.4 (4.7)	9.7 (5.1)	0.5	<0.001	<0.001

**Table 4:** Multiple linear regression analysis in each country.

Psychosocial Variables by Demographic Variables and Time		Norway		USA		UK		AUS	
		B (SE)	*p*	B (SE)	*p*	B (SE)	*p*	B (SE)	*p*
**GHQ**									
	Gender (women)	1.33 (0.67)	0.047	1.24 (0.4)	0.002	1.93 (0.65)	0.003	1.54 (1.24)	0.213
	Age	−0.99 (0.24)	<0.001	−0.69 (0.14)	<0.001	−0.38 (0.21)	0.065	−0.26 (0.4)	0.514
	Civil status (married/cohabiting)	−1.48 (0.59)	0.012	0.34 (0.38)	0.373	−2.37 (0.51)	<0.001	−1.52 (1.11)	0.17
	Children (yes)	−1.09 (0.68)	0.113	−0.62 (0.39)	0.115	0.72 (0.77)	0.352	−2.01 (1.27)	0.113
	Education (Master’s or above)	0.89 (0.57)	0.12	−0.46 (0.36)	0.193	−0.3 (0.66)	0.653	−2.39 (1.06)	0.024
	Employment (part-time)	1.16 (0.63)	0.066	0.28 (0.43)	0.514	0.81 (0.54)	0.133	2.29 (0.97)	0.018
	Place of work (healthcare as ref.)								
	Other	1.08 (0.71)	0.13	0.58 (0.46)	0.204	1.02 (0.6)	0.086	1.71 (1.11)	0.126
	Public safety, police, fire services, etc.	0.35 (1.09)	0.75	−0.16 (0.62)	0.802	0.57 (0.77)	0.463	0.5 (1.5)	0.738
	Education	0.54 (0.88)	0.536	0.98 (0.53)	0.066	0.86 (0.83)	0.301	4.13 (1.89)	0.029
	Trade industry	−1 (1.13)	0.376	−0.55 (0.72)	0.447	2.77 (1.1)	0.012	1.56 (1.95)	0.426
	Living area (rural as ref.)	0.11 (0.44)	0.8	0.25 (0.27)	0.367	0.3 (0.36)	0.399	0.27 (1.06)	0.798
	Time (November vs. April)	1.83 (0.68)	0.007	0.02 (0.42)	0.962	1.15 (0.68)	0.09	−2.06 (1.14)	0.072
**CL**									
	Gender (women)	−0.1 (0.26)	0.714	−0.17 (0.16)	0.267	−0.14 (0.21)	0.511	1.05 (0.44)	0.016
	Age	0.29 (0.09)	0.003	0.11 (0.06)	0.039	0.03 (0.07)	0.674	0.01 (0.14)	0.933
	Civil status (married/cohabiting)	0.66 (0.23)	0.005	−0.15 (0.15)	0.331	0.59 (0.17)	0.001	0.48 (0.39)	0.227
	Children (yes)	0.34 (0.27)	0.213	0.2 (0.16)	0.193	0.15 (0.25)	0.55	0.01 (0.45)	0.994
	Education (Master’s or above)	0.11 (0.22)	0.628	−0.09 (0.14)	0.53	−0.05 (0.22)	0.821	−0.03 (0.38)	0.94
	Employment (part-time)	0.06 (0.25)	0.806	0.36 (0.17)	0.035	0.14 (0.18)	0.43	0 (0.34)	0.993
	Place of work (healthcare as ref.)								
	Other	−0.25 (0.28)	0.381	−0.19 (0.19)	0.305	−0.16 (0.2)	0.426	0.39 (0.39)	0.322
	Public safety, police, fire services, etc.	−0.24 (0.43)	0.58	−0.46 (0.24)	0.059	0.02 (0.26)	0.943	0.47 (0.53)	0.377
	Education	−0.06 (0.34)	0.861	−0.09 (0.21)	0.684	−0.19 (0.27)	0.479	−0.52 (0.67)	0.434
	Trade industry	0.58 (0.44)	0.184	−0.48 (0.29)	0.102	−0.48 (0.36)	0.18	0.74 (0.7)	0.295
	Living area (rural as ref.)	0 (0.18)	0.996	0.05 (0.11)	0.626	−0.09 (0.12)	0.456	0.16 (0.37)	0.672
	Time (November vs. April)	0.09 (0.29)	0.756	0.12 (0.17)	0.463	0.12 (0.22)	0.599	−0.61 (0.41)	0.134
**PSW**									
	Gender (women)	0.15 (0.08)	0.065	0.12 (0.05)	0.012	0.13 (0.07)	0.082	0.18 (0.15)	0.214
	Age	−0.1 (0.03)	0.001	−0.11 (0.02)	<0.001	−0.06 (0.02)	0.007	−0.07 (0.05)	0.113
	Civil status (married/cohabiting)	−0.34 (0.07)	<0.001	−0.14 (0.05)	0.003	−0.46 (0.06)	<0.001	−0.26 (0.13)	0.043
	Children (yes)	−0.17 (0.08)	0.038	−0.1 (0.05)	0.034	0.03 (0.09)	0.761	−0.24 (0.15)	0.101
	Education (Master’s or above)	−0.04 (0.07)	0.572	−0.09 (0.04)	0.055	−0.04 (0.07)	0.621	−0.28 (0.12)	0.027
	Employment (part-time)	0.14 (0.08)	0.076	0.06 (0.05)	0.239	0.08 (0.06)	0.183	0.28 (0.11)	0.014
	Place of work (healthcare as ref.)								
	Other	0.1 (0.09)	0.26	0.07 (0.06)	0.193	0.09 (0.07)	0.192	0.05 (0.13)	0.724
	Public safety, police, fire services, etc.	0.11 (0.13)	0.395	0.08 (0.08)	0.28	0.01 (0.09)	0.897	−0.12 (0.18)	0.481
	Education	0.02 (0.11)	0.857	0.14 (0.07)	0.031	0.04 (0.09)	0.704	0.46 (0.22)	0.04
	Trade industry	−0.01 (0.14)	0.918	0.06 (0.09)	0.505	0.21 (0.12)	0.096	0.21 (0.23)	0.356
	Living area (rural as ref.)	−0.02 (0.05)	0.691	0.01 (0.03)	0.78	−0.01 (0.04)	0.804	−0.05 (0.12)	0.715
	Time (November vs. April)	0.23 (0.08)	0.006	−0.01 (0.05)	0.889	0.19 (0.08)	0.011	−0.31 (0.13)	0.02
**Loneliness**									
	Gender (women)	0.47 (0.49)	0.337	−0.13 (0.31)	0.664	−0.43 (0.41)	0.297	−0.77 (0.87)	0.376
	Age	−0.36 (0.18)	0.045	−0.33 (0.11)	0.003	−0.2 (0.13)	0.131	−0.26 (0.28)	0.35
	Civil status (married/cohabiting)	−2.23 (0.43)	< 0.001	−0.86 (0.3)	0.004	−2.41 (0.32)	< 0.001	−0.76 (0.78)	0.327
	Children (yes)	−0.84 (0.5)	0.097	−0.26 (0.31)	0.396	−0.56 (0.5)	0.264	−0.04 (0.89)	0.962
	Education (Master’s or above)	−0.08 (0.42)	0.847	−0.79 (0.28)	0.004	0.04 (0.42)	0.915	−1.66 (0.74)	0.026
	Employment (part-time)	0.54 (0.46)	0.242	0.2 (0.34)	0.552	0.75 (0.35)	0.031	0.68 (0.68)	0.314
	Place of work (healthcare as ref.)								
	Other	0.56 (0.52)	0.284	0.43 (0.36)	0.231	0.43 (0.38)	0.257	0.35 (0.78)	0.657
	Public safety, police, fire services, etc.	0.93 (0.8)	0.245	0.68 (0.49)	0.165	0.47 (0.49)	0.339	0.01 (1.05)	0.997
	Education	−0.28 (0.65)	0.666	0.54 (0.41)	0.193	0.36 (0.53)	0.501	3.12 (1.32)	0.018
	Trade industry	−0.57 (0.84)	0.497	0.03 (0.57)	0.96	1.35 (0.71)	0.057	3.94 (1.37)	0.004
	Living area (rural as ref.)	−0.16 (0.32)	0.612	−0.1 (0.21)	0.627	−0.20 (0.23)	0.381	0.36 (0.74)	0.627
	Time (November vs. April)	0.71 (0.5)	0.157	0.34 (0.33)	0.301	0.64 (0.44)	0.145	−1.07 (0.8)	0.18

## Data Availability

The data presented in this study are available upon request from the corresponding author on completion of the research. The data are not published/available due to ongoing publications stemming from the project and different data protection regulations in the four countries involved in the study.
